# Protective Effects of Hydrogen Sulfide Against the ATP-Induced Meningeal Nociception

**DOI:** 10.3389/fncel.2020.00266

**Published:** 2020-09-02

**Authors:** Kseniia Koroleva, Elizaveta Ermakova, Alsu Mustafina, Raisa Giniatullina, Rashid Giniatullin, Guzel Sitdikova

**Affiliations:** ^1^Department of Human and Animal Physiology, Institute of Fundamental Medicine and Biology, Kazan Federal University, Kazan, Russia; ^2^A.I. Virtanen Institute for Molecular Sciences, Faculty of Health Sciences, University of Eastern Finland, Kuopio, Finland

**Keywords:** migraine, trigeminal nerve, mast cells, ATP, H_2_S, P2X3 receptor

## Abstract

We previously showed that extracellular ATP and hydrogen sulfide (H_2_S), a recently discovered gasotransmitter, are both triggering the nociceptive firing in trigeminal nociceptors implicated in migraine pain. ATP contributes to meningeal nociception by activating the P2X3 subunit-containing receptors whereas H_2_S operates mainly *via* TRP receptors. However, H_2_S was also proposed as a neuroprotective and anti-nociceptive agent. This study aimed to test the effect of H_2_S on ATP-mediated nociceptive responses in rat meningeal afferents and trigeminal neurons and on ATP-induced degranulation of dural mast cells. Electrophysiological recording of trigeminal nerve activity in meninges was supplemented by patch-clamp and calcium imaging studies of isolated trigeminal neurons. The H_2_S donor NaHS induced a mild activation of afferents and fully suppressed the subsequent ATP-induced firing of meningeal trigeminal nerve fibers. This anti-nociceptive effect of H_2_S was specific as an even stronger effect of capsaicin did not abolish the action of ATP. In isolated trigeminal neurons, NaHS decreased the inward currents and calcium transients evoked by activation of ATP-gated P2X3 receptors. Moreover, NaHS prevented ATP-induced P2X7 receptor-mediated degranulation of meningeal mast cells which emerged as triggers of migraine pain. Finally, NaHS decreased the concentration of extracellular ATP in the meningeal preparation. Thus, H_2_S exerted the multiple protective actions against the nociceptive effects of ATP. These data highlight the novel pathways to reduce purinergic mechanisms of migraine with pharmacological donors or by stimulation production of endogenous H_2_S.

## Introduction

Long-lasting migraine pain likely originating from meninges, involves local inflammation, sensitization and activation of trigeminal afferents by multiple endogenous compounds released from local vessels, somatic and parasympathetic nerves and various immune cells such as mast cells occupying this area (Bolay et al., 2002; Levy et al., 2007; Olesen et al., 2009; Burstein et al., 2015; Koroleva et al., 2019). To find new pharmacological interventions in migraine, much attention is currently paid to the substances triggering pain such as neuropeptides, most notably, calcitonin gene-related peptide (CGRP) which promote neurogenic inflammation in meninges (Ebersberger et al., 1999; Lassen et al., 2002; Schou et al., 2017) or gaseous transmitter nitric oxide (NO) promoting pain and dilating cortical vessels (Reuter et al., 2001; Pryazhnikov et al., 2014; Messlinger et al., 2020). Less interest is given to endogenous molecules which can exhibit the anti-nociceptive and anti-inflammatory effects in migraine.

Hydrogen sulfide (H_2_S) is a common gaseous transmitter that regulates many physiological and pathological processes (Kimura, 2011; Hermann et al., 2012; Wang, 2012; Paul and Snyder, 2018). In mammals, H_2_S is generated from sulfur-containing amino acids, primarily L-cysteine and L-homocysteine by the enzymes cystathionine-β-synthase (CBS), cystathionine-γ-lyase (CSE) and 3-mercaptopyruvate sulfurtransferase (Kimura, 2011; Hermann et al., 2012; Wang, 2012; Paul and Snyder, 2018). CBS is the main enzyme for the synthesis of H_2_S in the nervous tissue, whereas, in the cardiovascular system, liver, and kidneys, the main synthesis enzyme for H_2_S is CSE (Kimura, 2011).

Emerging data suggest the participation of this endogenous gaseous transmitter in nociception (Bhatia et al., 2005; Cunha and Verri, 2007; Okubo et al., 2012; Xu et al., 2019). Immunohistochemical studies have shown expression of the H_2_S synthesis enzyme, CBS in sensory DRG and trigeminal ganglia (Xu et al., 2009; Feng et al., 2013). Moreover, the level of CBS expression increases with the development of inflammation (Bhatia, 2015). Notably, inflammation in meninges essentially contributes to sensitization related to migraine pain (Strassman et al., 1996; Ebersberger et al., 1999; Waeber and Moskowitz, 2005). However, the available data on the role of H_2_S in the nociceptive system is contradictory (Bhatia et al., 2005; Cunha and Verri, 2007; Kida et al., 2015; Chen et al., 2019; Xu et al., 2019). H_2_S can activate several pro-nociceptive receptors such as the TRPV1, and TRPA1 receptors (Trevisani et al., 2005; Teicher et al., 2017; Pozsgai et al., 2019; Roa-Coria et al., 2019). On the other hand, H_2_S activates ATP-dependent and Ca^2+^-activated potassium channels which can reduce the neuronal excitability through membrane hyperpolarization (Sitdikova et al., 2010; Mustafina et al., 2015).

This gaseous transmitter also stabilizes mast cells (Roviezzo et al., 2015; Rodrigues et al., 2017; Matsui et al., 2019). Also, recent studies propose the antioxidant and anti-inflammatory potential of H_2_S (Chen et al., 2019; Melo et al., 2019; Yakovleva et al., 2020; Yurinskaya et al., 2020).

Recently, we showed that the H_2_S donor NaHS can trigger nociceptive firing in rat trigeminal afferents (Koroleva et al., 2017). However, this pro-nociceptive effect was transient as the firing quickly returned to the baseline level in the presence of H_2_S.

Likewise, we have also shown that ATP stimulates the rat and mouse trigeminal afferents thus, exerting a clear pro-nociceptive and prolonged effect *via* the P2X3 subunit-containing receptors (Yegutkin et al., 2016; Koroleva et al., 2019). Moreover, ATP degranulated meningeal mast cells releasing serotonin, which indirectly supports the pro-nociceptive effect of purinergic agonists (Koroleva et al., 2019). In general, ATP activated P2X3 receptors are expressed in ~80% of trigeminal neurons (Fabbretti et al., 2006) suggesting the leading role of ATP signaling in trigeminal nociception.

As two pro-nociceptive agents, ATP and H_2_S can be endogenously generated in the trigeminovascular system, the unsolved issue remains whether they can interact. The interaction of classical transmitters with gasotransmitters is a relatively novel little-explored field. Co-expression of H_2_S producing enzyme CBS and ATP-activated P2X receptors have been detected in sensory ganglia (Xu et al., 2009). This fact suggests a possible interaction between signaling cascades activated by H_2_S and ATP *via* P2X receptors. However, data on the effect of H_2_S on purinergic mechanisms of trigeminal nociception, in particular, in migraine are lacking.

The present study aimed to explore the modulatory action of H_2_S on the pronociceptive effects of ATP in the trigeminal nerve, in isolated trigeminal neurons and on meningeal mast cells. We report that the mild and transient activation effect of H_2_S is followed by almost completed suppression for ATP pro-nociceptive signaling including neurons and mast cells.

## Materials and Methods

### Animals

The experiments with cultured trigeminal neurons were carried out on male P9–12 Wistar rats, whereas 4–6 weeks rats were used for testing hemiskull preparation and meningeal mast cell staining. Animals from the Animal Center of the University of Eastern Finland (Kuopio) and vivarium of Kazan Federal University were used. Rats were housed in cages with controlled temperature and humidity and a 12-h light cycle. Food and water were provided *ad libitum*. The experimental protocols complied with the ethical standards for the humane treatment of animals adopted at the Kazan Federal University and approved by the Local Ethics Committee of KFU (protocol No. 8 dated 05.05.2015), and complied with the Council of the European Union Directive of September 22, 2010 (2010/63/EEC) and were approved by the Committee for the Use of Animals of the University of Eastern Finland (licenses EKS-004-2014 and EKS-002-2017). All measures were taken to minimize the number of animals used in the experiments.

### Objects of Study

#### Hemiskull Preparation

To study meningeal nociception, we used the isolated rat hemiskull with intact innervation of dura mater (De Col et al., 2012). This model of the peripheral nociceptive processes in the dura mater allows most directly to assess the neurochemical mechanisms of migraine pain generation (Zakharov et al., 2015). After decapitation, the rat skull was carefully cleaned from all cranial muscles. Then the skull was divided along the sagittal section into two halves (hemiskulls), whereas the brain was gently removed from the cranium. The meningeal processes of the mandibular branch of the trigeminal nerve called “*nervus spinosus,*” that innervates the receptive field around the middle meningeal artery (MMA), were isolated to be placed inside the recording electrode. The hemiskulls were stabilized in artificial cerebrospinal fluid (ACSF) of the following composition (in mM): 120 NaCl, 2.5 KCl, 2 CaCl_2_, 1 MgCl_2_, 11 glucose, 24 NaHPO_4_, 30 NaHCO_3_ with constant oxygenation of 95% O_2_/5% CO_2_ for at least 30 min before starting the experiment.

#### Trigeminal Culture

After rat decapitation, the trigeminal ganglia were extracted, placed in a cold Ham’s F12 nutrient mixture, and then were chopped. Cells dissociation was carried out in an enzymatic cocktail containing 0.25 mg/ml trypsin, 1 mg/ml collagenase and 0.2 mg/ml DNAse at shaker at a temperature of 37°C, at 1,000 rpm, for 25 min. Dissociated cells were placed on coverslips coated with poly-L-lysine and were kept in an incubator at a temperature of 37°C, in 5% CO_2_ for 24 h before the start of the experiments.

### Electrophysiology

#### The Activity of the Trigeminal Nerve Peripheral Branch

The isolated hemiskulls were fixed in the experimental chamber provided with a flow perfusion system. Drugs were applied with a speed of 6–7 ml/min. Under visual control, the peripheral process of the nervus spinosus was sucked into a glass electrode (tip diameter of ~150 μm). The application of substances was performed to the receptive field around the area of divergence of MMA (Schueler et al., 2014). The isolated preparation was washed with ACSF under constant oxygenation with 95% O_2_/5% CO_2_, the pH was maintained at 7.20–7.35. Electrical signals were recorded using a DAM80 amplifier (World Precision Instruments, Sarasota, FL, USA). The signals were digitized on a PC using the NI PCI6221 board (National Instruments, Austin, TX, USA) and WinEDR v.3.2.7 software (Strathclyde University, UK). The two-phase signals, with duration in the range of 0.3–1.5 ms were considered as action potentials. Data recorded immediately before the application of the substance was used as a control.

#### P2X3 Receptor Responses in Trigeminal Ganglion Neurons

During the experiment, cells were constantly perfused with a solution containing (mM): 148 NaCl, 5 KCl, 1 MgCl_2_, 2 CaCl_2_, 10 HEPES, 10 D-Glucose, pH 7.2. The intrapipette solution contained (mM): 145 KCl, 1 MgCl_2_, 10 HEPES, 5 EGTA, 0.5 CaCl_2_, 2 Mg-ATP, 0.5 Na-GTP, 5 KCl, pH 7.2. The solution was supplied using a gravity-controlled perfusion system (ALA Scientific Instruments Westbury, Farmingdale, NY, USA). P2X3-induced currents were recorded using whole-cell patch-clamp with Axopatch-200B amplifiers (Axon Instruments/Molecular Devices, San Jose, CA, USA) and borosilicate glass patch pipettes (Harvard Apparatus, Holliston, MA, USA) with a resistance of 3–10 MΩ. P2X3 currents were induced by local application of the agonist α,β-meATP at a concentration of 20 μM for 2 s using a rapid perfusion system (Rapid Solution Changer 200, BioLogic Science Instruments, France), with a solution supply of ~20 ms. To prevent desensitization of P2X3 receptors, α, β-meATP was applied at intervals of 5 min. Patch-clamp data were analyzed using the Clampfit software (Axon Instruments/Molecular Devices, San Jose, CA, USA).

#### Calcium Imaging in Trigeminal Cells

To visualize calcium signals trigeminal cells were loaded with Fluo4-AM fluorescent marker (2 μM) at 37°C for 30–40 min in dark following by washout with an extracellular solution for 10 min. Fluorescent visualization of stained cells was carried out using an Axio Observer D1 microscope (Carl Zeiss, Germany). Fluorescence images were recorded using an AxioCam MRm high-speed camera (Carl Zeiss, Germany). The test substances were applied using a gravity-controlled perfusion system (ALA Scientific Instruments Westbury, NY, USA). To differentiate neuronal cells from glia, a 100 mM KCl solution was applied at the end of each experiment. Image processing software (NIH, Bethesda, MD, USA) was used to process fluorescence images and estimate fluorescence intensity (in arbitrary units, a.u.). Peak amplitude was calculated using the MATLAB software package (The MathWorks, Novi, MI, USA).

#### Mast Cell Degranulation

To study meningeal mast cell degranulation, we stained meninges (Wistar rats P35–40) with Toluidine Blue (Guselnikova et al., 2014). Hemiskulls were filled with the studied solutions for 20 min, and then they were fixed in paraformaldehyde (4%) for 12 h. Before isolation of the meninges, the hemiskulls were washed in a phosphate-saline buffer solution of the following composition (mM): 137 NaCl, 2.7 KCl, 10 Na_2_HPO_4_, 1.8 KH_2_PO_4_. Isolated dura mater was placed on a glass slide. Staining with Toluidine Blue lasted 10 min, after three times washing with PBS the preparations were fixed with ethanol (95–99%). Pictures from stained meninges were taken at 20× magnification on Olympus AX70 microscope (Tokyo, Japan). Mast cells with inhomogeneous staining, pale cells, and cells with disfigured borders surrounding positively stained granules were ranked as degranulated (Shelukhina et al., 2017). The degranulation was evaluated in a blind manner approaching 100 cells from 10 different fields (20×) from the meningeal preparation in each experiment. The rate of degranulation was calculated as a % of the degranulated cells to the total number of cells (Pedersen et al., 2015; Shelukhina et al., 2017; Koroleva et al., 2019).

### Measuring the Extracellular Level of ATP in the Dura Mater

The concentration of ATP released from the rat dura mater (Wistar rats, P35–45) was estimated using the ATP luminescence analysis kit (PerkinElmer, Waltham, MA, USA). Isolated rat hemiskulls were filled with solutions. After 20 min incubation, 100 μl of the media were taken away for analysis. The analysis was performed according to the ATP lite kit protocol using 96-well plates (Costar, Corning, USA). Luminescence was measured using a POLARstar Optima microplate reader (BMG Labtech GmbH, Germany).

### Chemicals

The agonists α,β-meATP (20 μM), adenosine triphosphate (ATP, 100 μM), BzATP triethylammonium salt (BzATP, 30 μM), oxidized glutathione (GSSG, 1 mM), capsaicin (1 μM, all from Sigma–Aldrich, St. Louis, MO, USA) were used. Toluidine Blue (Sigma–Aldrich, St. Louis, MO, USA) was employed for labeling mast cells. Sodium hydrosulfide (NaHS, Sigma–Aldrich, St. Louis, MO, USA) was used as a hydrogen sulfide donor. In solutions, NaHS dissociates into HS^−^ ions and binds to the hydrogen proton H^+^ to form H_2_S. H_2_S concentration in solution depends on the temperature, pH, and ionic strength (Whitfield et al., 2008; Nagy et al., 2014). In the bath solution (pH 7.4) at 20°C, about 22% of total sulfide is expected to be as free H_2_S (Sitdikova et al., 2014). Real-time measurements of H_2_S concentration using amperometry indicated a fast loss of the sulfide after the volatilization of H_2_S (DeLeon et al., 2012; Sitdikova et al., 2014). In our experiments, NaHS was used at a concentration of 100 μM, which provides approximately 11 μM H_2_S in the perfusion system. NaHS stock solutions were prepared immediately before the experiment and they were kept tightly closed in a dark place until use.

### Statistical Analysis

Statistical data processing was performed using MATLAB and Origin Pro 2015 software (OriginLab, Northampton, MA, USA). To assess reliability, the Student’s *t*-test was used (for paired and independent samples). All values are indicated as the mean ± standard error of the mean (M ± SEM). In our study, *n* indicates the number of animals. Differences were considered statistically significant at *p* < 0.05.

## Results

### NaHS Counteracted the Pro-nociceptive Effect of ATP in the Meningeal Trigeminal Nerve

**Figure 1:**
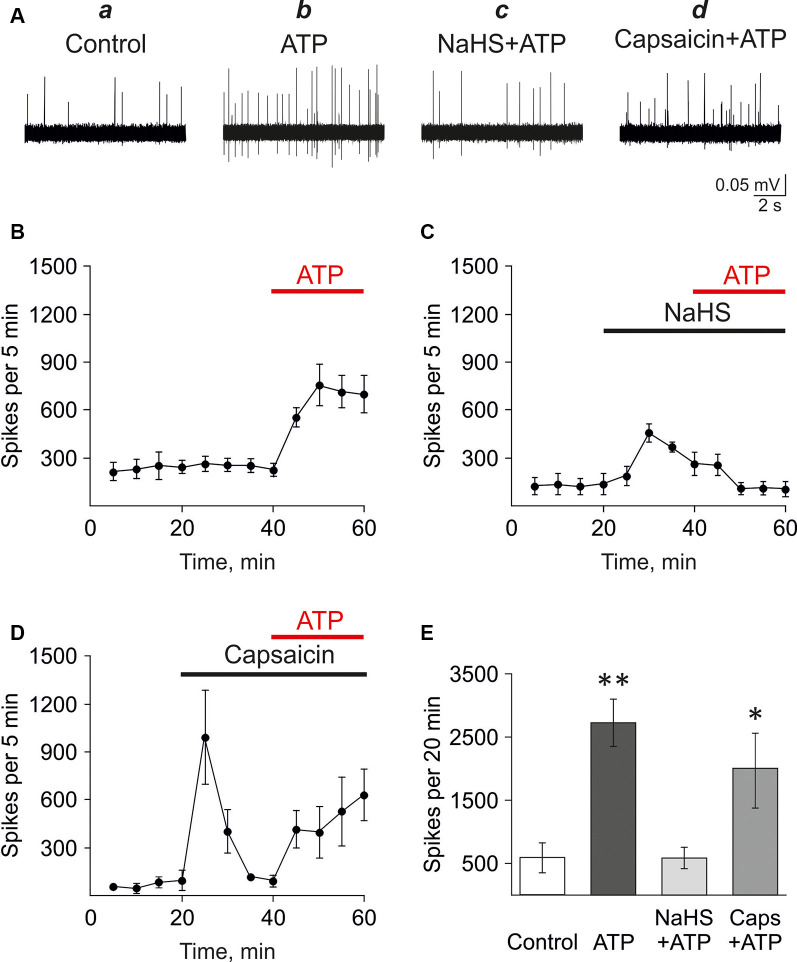
Testing the action of hydrogen sulfide (H_2_S) on the pro-nociceptive effect of ATP in trigeminal afferents. **(A)** Example traces of action potentials in the trigeminal nerve in control **(a)**, with ATP application (100 μM; **b**), ATP plus NaHS application after preincubation in NaHS (100 μM; **c**), ATP plus capsaicin application after preincubation in capsaicin (1 μM; **d**). **(B)** The frequency of action potentials during application of ATP (*n* = 5). **(C)** The frequency of action potentials during application of ATP (100 μM) in the presence of NaHS after preincubation with NaHS (100 μM; *n* = 4). **(D)** The frequency of action potentials during the application of ATP (100 μM) in the presence of capsaicin after preincubation with capsaicin (1 μM; *n* = 4). **(E)** The histograms showing the frequency of action potentials per 20 min in the trigeminal nerve in control and after application of ATP (100 μM), α,β-meATP (20 μM), in control and in the presence of NaHS or capsaicin. *p < 0.05, **p < 0.01.

First, we analyzed the effect of H_2_S on the pro-nociceptive effect of ATP in the rat trigeminal meningeal nerve after the incubation of the hemiskull preparation in a solution containing the H_2_S donor NaHS. In control, the application of ATP (100 μM) induced a strong and prolonged surge in the activity of the rat trigeminal nerve. Thus, the frequency of action potentials increased from 227.8 ± 38.0 spikes per 5 min in control up to 755.8 ± 129.4 spikes per 5 min after 10 min of ATP application (*n* = 5, *p* < 0.05; Figures 1Aa,b,B). During 20 min application of ATP, 2,725.6 ± 374.2 spikes were comparing with 588.5 ± 236.0 spikes for the same period in control (*n* = 5; *p* < 0.05; Figure 1E). Application of the H_2_S donor NaHS (100 μM) elicited only a transient increase of trigeminal nerve spiking (from 139.6 ± 65.4 to 458.0 ± 56.1 spikes per 5 min; *n* = 4; *p* < 0.05) which further declined in the presence of NaHS to the baseline level. The subsequent application of ATP (100 μM) combined with 100 μM NaHS did not increase the frequency of action potentials which was 109.5 ± 39.9 spikes per 5 min after 10 min action of this purinergic agonist (Figures 1Ac,C). This activity (586.7 ± 168.5 spikes per 20 min) in the presence of NaHS+ATP did not differ from the basal frequency of action potentials (*n* = 4, *p* > 0.05; Figure 1E).

Next, the H_2_S scavenger oxidized glutathione (GSSG; Pei et al., 2011) was used to probe that the inhibition of ATP-induced nociceptive response was mediated by H_2_S. GSSG (1 mM) by itself did not affect the frequency of action potentials. Thus, there were 723.3 ± 138.1 spikes per 5 min in control and 722.5 ± 147.8 spikes per 5 min by 20 min of GSSG application (*n* = 3; Supplementary Figure S1). ATP (100 μM) increased action potentials frequency to 1,695.3 ± 481.4 spikes per 5 min by 10 min of application of (*n* = 3, *p* < 0.05, Supplementary Figures S1A,C). In the presence of 1 mM GSSG NaHS (100 μM) did not increase the frequency of action potentials (Supplementary Figures S1B,D). Thus, there were 810.0 ± 134.3 spikes per 5 min in control and 918.3 ± 238.9 spikes per 5 min in glutathione + NaHS. Application of ATP (100 μM) increased spiking activity up to 2,258.0 ± 558.28 spikes per 5 min (*n* = 3, *p* < 0.05) similar to the effects of ATP in control (Supplementary Figures S1B,D).

We have shown previously that P2X3 receptors mediated the pro-nociceptive effects of ATP in the trigeminal nerve (Yegutkin et al., 2016). Therefore, we analyzed the effects of NaHS application on the nociceptive firing induced by the P2X3 receptor agonist α,β-meATP. In control, α,β-meATP (20 μM) similarly to ATP, increased the frequency of action potentials from 545.6 ± 268.8 spikes per 5 min in control up to 957.3 ± 51.1 spikes per 5 min after 10 min (*n* = 3, *p* < 0.05). The preliminary application of NaHS (100 μM) prevented an increase of P2X3 mediated response. Thus, the frequency of action potentials was only 381.7 ± 125.2 spikes per 5 min after 10 min action of α,β-meATP compared to control (561.2 ± 283.1, *n* = 3, *p* > 0.05).

These data revealed the inhibitory action of H_2_S on ATP-induced activation of P2X3 receptors in the trigeminal nerve endings in the meninges.

As we (Koroleva et al., 2017) and others (Roa-Coria et al., 2019) showed that the action of H_2_S is mediated, at least, partly by TRPV1 receptors, it could be that this inhibition is due the negative crosstalk between TRPV1 and P2X3 receptors. Thus, it has been shown previously in isolated neurons that the activation of TRPV1 receptors decreased the subsequent activation of ATP-gated P2X3 receptors (Stanchev et al., 2009). We hypothesized that a similar mechanism might underlie the inhibitory action of H_2_S on ATP signaling in the meninges. Therefore, the effect of ATP was analyzed in the hemiskull preparation after the preliminary activation of TRPV1 with the specific agonist capsaicin. In control, the frequency of action potentials was 85.0 ± 32.9 spikes per 5 min (*n* = 4). The application of capsaicin (1 μM) increased activity to 994.0 ± 294.9 spikes per 5 min (*n* = 4; *p* < 0.05) followed, like with NaHS, by a subsequent decline in the frequency of action potentials to values close to control (119.0 ± 12.0 spikes), likely due to desensitization of the TRPV1 receptors. However, unlike NaHS, the subsequent application of ATP (100 μM) in the presence of capsaicin efficiently increased the frequency of action potentials to 414.0 ± 117.9 spikes per 5 min (*n* = 4; *p* < 0.05) at 10 min and 527.6 ± 217.4 spikes per 5 min at 20 min (*n* = 4; *p* > 0.05; Figures 1Ad,D). This effect was comparable with the action of ATP alone. Thus, the application of ATP, combined with capsaicin, generated 1,969.3 ± 605.7 spikes per 20 min which were not significantly different from ATP alone (2,725.6 ± 374.2 spikes per 20 min; *p* > 0.05; Figure 1E).

These results indicated that the suppressing action of NaHS on the pro-nociceptive effect of ATP was specific and was not shared by the agonist of TRPV1 receptors capsaicin.

### NaHS Decreased P2X3-Mediated Currents and Calcium Transients in Trigeminal Neurons

**Figure 2:**
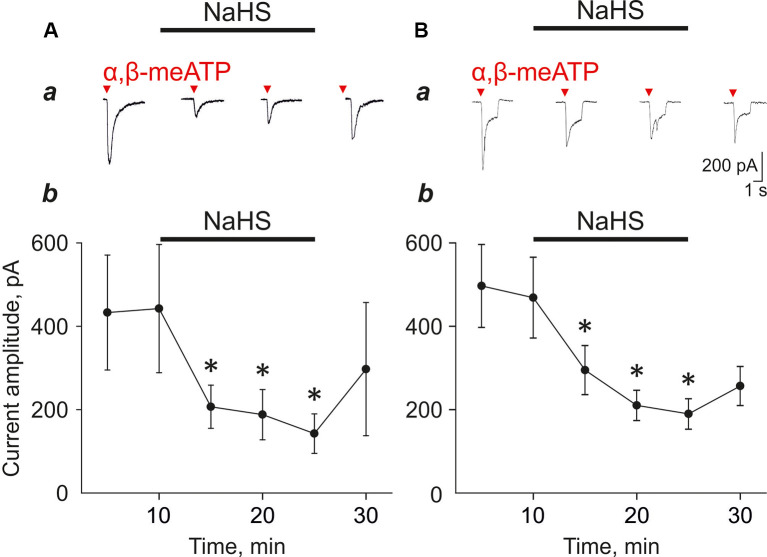
Effect of NaHS (100 μM) on α,β-meATP induced currents in trigeminal ganglion neurons. **(A)** Examples of fast type currents in control and in the presence of NaHS **(a)**. Changes in the amplitude of the fast type currents (**b**, *n* = 5 cells). **(B)** Examples of mixed type currents in trigeminal ganglion neurons in control and in the presence of NaHS **(a)**. Changes in the amplitude of the fast component of the mixed type currents (**b**, *n* = 9 cells). *p < 0.05.

To further study the mechanisms of the inhibitory effect of NaHS on the pro-nociceptive properties of ATP we used the isolated trigeminal ganglion neurons. In control, the application of α,β-meATP (20 μM) agonist of the P2X3 receptor-induced fast, slow, and mixed currents, consisting of two components with fast and slow desensitization (Figures 2Aa,Ba). It is known that, in sensory neurons, the P2X2 and P2X3 subunits can form homomeric P2X2, homomeric P2X3, or heteromeric P2X2/3 receptors (Lewis et al., 1995; Kowalski et al., 2015). P2X3 receptors account for fast currents, whereas slow and mixed currents are mediated by heteromeric receptors co-expressing P2X3 and P2X2 subunits (Lewis et al., 1995; Kowalski et al., 2015).

To test the action of H_2_S on P2X3 receptors we focused on fast and mixed currents as we noted in this and in previous experiments that the slow component is relatively unstable during repetitive agonist applications. Application of NaHS (100 μM) decreased the amplitude of the fast currents from 442.4 ± 164.0 pA to 206.9 ± 51.7 pA (five cells; *n* = 5; *p* < 0.05; Figure 2Ab) and the amplitude of the mix currents from 468.7 ± 96.9 to 294.8 ± 58.9 (nine cells; *n* = 8; *p* < 0.05; Figure 2Bb) by 5 min of NaHS application. Washout only partially recovered responses. To minimize the effect possible desensitization of P2X3 receptors, we performed repeated application of α,β-meATP (20 μM) with a 5 min interval in control and did not reveal the decrease of the current amplitudes. The amplitude of currents during first application was 879.22 ± 242.92 pA, second—1,071.43 ± 329.67 pA, third—1,043.14 ± 315.22 pA, fourth—1,190.30 ± 351.9 pA and fifth—1,037.17 ± 314.88 pA (seven cells; *n* = 4, Supplementary Figures S2A,B).

**Figure 3:**
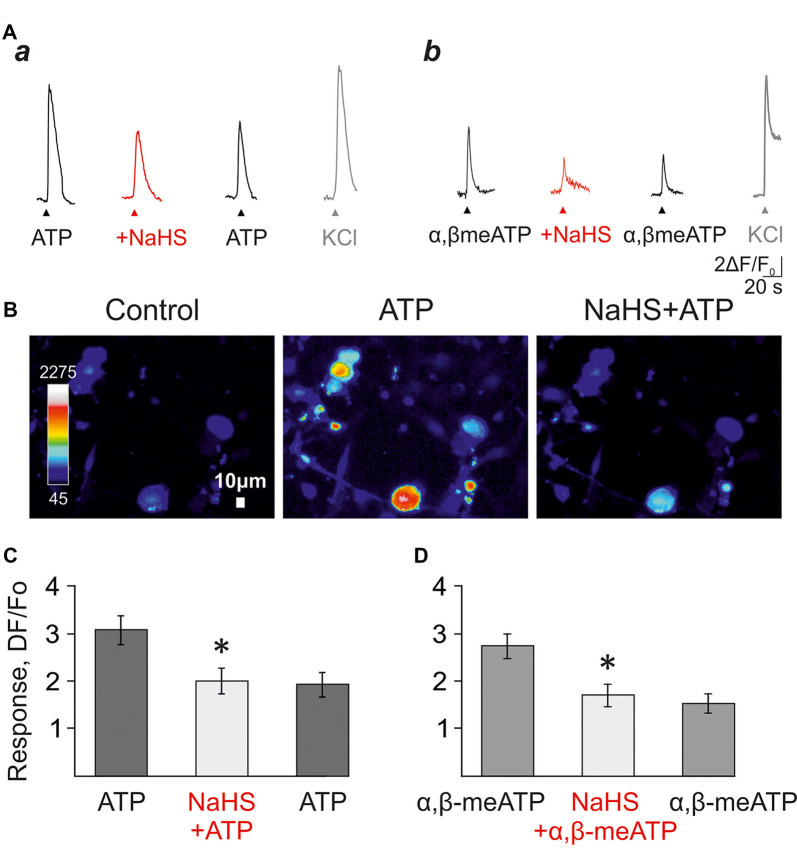
The effect of NaHS on ATP-induced calcium signals in isolated trigeminal neurons. **(A)** Examples of calcium signals induced by application of 100 μM ATP **(a)**, or 20 μM α,β-meATP **(b)** in control, and in the presence of 100 μM NaHS. KCl was applied to distinguish neurons from trigeminal glial cells. **(B)** Pseudo-color images showing ATP-induced calcium signals in control and after the preliminary application of NaHS. **(C)** Histograms showing the amplitudes of calcium signals in response to the application of ATP (100 μM) in control, and in the presence of NaHS (*n* = 66 cells). **(D)** Histograms showing the amplitudes of calcium signals in response to applications of α,β-meATP (20 μM) in control, and in the presence of NaHS (*n* = 27 cells). *p < 0.05.

In the next set of experiments, to test the action of H_2_S on unpatched neurons, we analyzed the effects of NaHS on calcium transients induced by ATP and α,β-meATP (Figure 3) in isolated trigeminal neurons. ATP (100 μM) responses were observed in 70% of the whole population of cells (61/87 cells) whereas α,β-meATP triggered calcium responses were observed in 41% of cells (33/136 cells).

In control, the repeated application of ATP (100 μM) or α, β-meATP (20 μM) with an interval of 5 min did not induce significant desensitization of calcium responses (Supplementary Figures S3A,B). The average amplitude of the first response to ATP was 3.53 ± 0.45 a.u., 3.42 ± 0.42 a.u. of the second and 2.93 ± 0.3 a.u. of the third response (61 cells; *n* = 3; *p* > 0.05; Supplementary Figures S3Aa,B). The application of NaHS (100 μM) decreased the amplitude of calcium response from 3.08 ± 0.3 a.u. to 2.01 ± 0.27 a.u. (66 cell; *n* = 3; *p* < 0.05; Figures 3Aa,B,C) and washout did not significantly change the average amplitude of the response (1.93 ± 0.26 a.u.).

The agonist α,β-meATP (20 μM) in control elicited calcium responses with an average amplitude of 3.53 ± 0.45 a.u.—the first application, 3.42 ± 0.42 a.u.—second and 2.92 ± 0.3 a.u. to the third one (33 cells; *n* = 3; *p* > 0.05; Supplementary Figures S3Ab,C). The application of NaHS (100 μM) decreased the amplitude of calcium responses from 2.73 ± 0.26 a.u. to 1.69 ± 0.23 a.u. (27 cells; *n* = 3, *p* < 0.05; Figures 3Ab,D) and washout did not significantly change the average amplitude of the response (1.52 ± 0.2 a.u.).

### NaHS Prevented Degranulation of Meningeal Mast Cells

**Figure 4:**
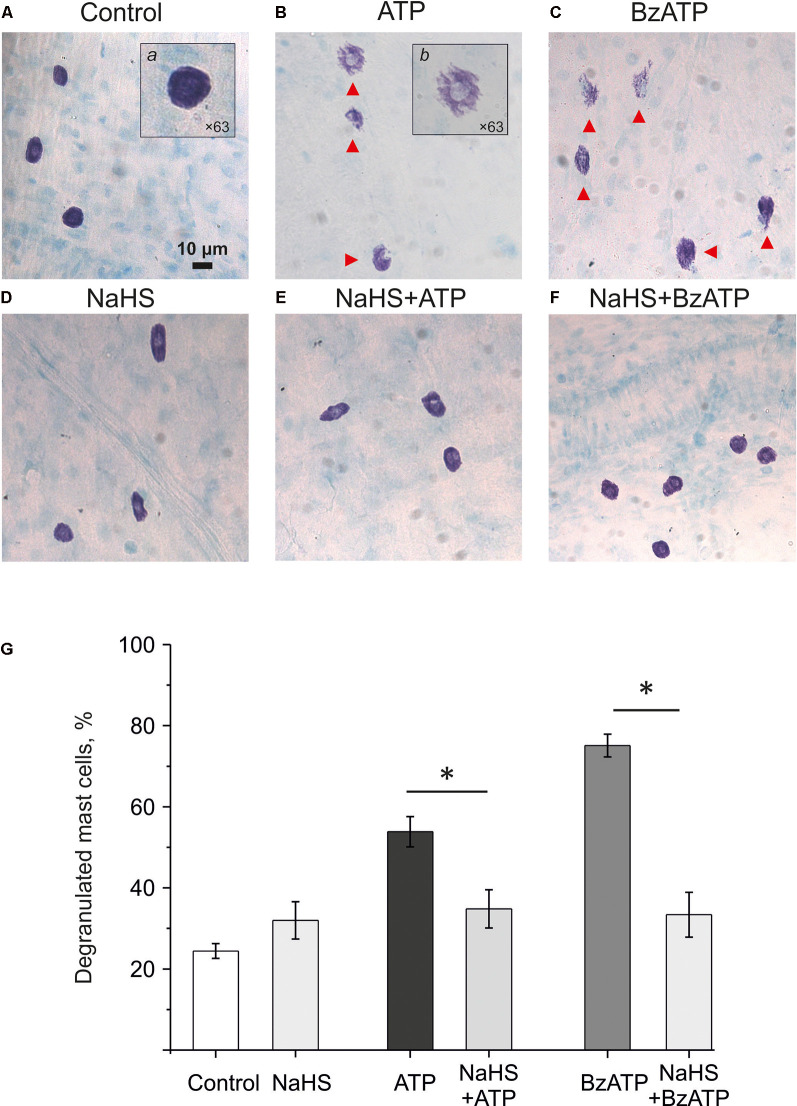
Effect of the H_2_S donor NaHS on ATP-induced mast cell degranulation. Images (×20) of Toluidin Blue stained rat meninges after incubation in basic solution **(A)** and after exposure to ATP (100 μM, **B**), BzATP (30 μM, **C**), NaHS (100 μM, **D**) or combination of NaHS+ATP **(E)** and NaHS+BzATP **(F)**. Notice red arrows indicating degranulated mast cells. Inserts **(a)** and **(b)** shows enlarged intact and degranulated mast cells (×63). **(G)** Histograms showing the percent of degranulated mast cells under various conditions (*n* = 6). *p < 0.05.

Mast cell degranulation in the meninges plays an important role in the pro-nociceptive effect of ATP by releasing endogenous nociceptive agent serotonin (Koroleva et al., 2019). Therefore, we assessed the potential protective effect of NaHS on ATP-induced mast cell degranulation. ATP (100 μM) caused a significant increase in the number of degranulated cells (53.8 ± 3.7%; *n* = 6; *p* < 0.05; Figures 4B,G) compared to the control group (24.4 ± 1.8%; *n* = 6; *p* < 0.05; Figures 4A,G). Incubation of meninges in a solution containing 100 μM NaHS for 30 min did not change the functional state of mast cells, as the number of degranulated cells (31.8 ± 4.6%; *n* = 6) did not significantly exceed the control values (Figures 4D,G). Notably, the pre-incubation in NaHS for 10 min followed by addition of ATP (100 μM) to the solution for 20 min also did not increase the number of degranulated cells (34.7 ± 4.7%; *n* = 6) indicating that H_2_S prevented mast cell degranulation by ATP (Figures 4E,G).

We previously showed that mast cell degranulation under the action of ATP is mediated by the activation of P2X7 receptors (Nurkhametova et al., 2019). Notably, this receptor is likely coupled to pannexin-1 channels, which can also transport ATP through the membrane (Iglesias and Spray, 2012; Kurashima et al., 2012; Wareham and Seward, 2016). Therefore, in subsequent experiments, we analyzed the ability of the P2X7 receptor agonist BzATP to activate meningeal mast cells in the presence of the H_2_S donor NaHS We found that 30 μM BzATP increased the number of degranulated cells to 75.14 ± 2.8% (*n* = 6, *p* < 0.05; Figures 4C,G). Pre-incubation in NaHS (100 μM) for 10 min prevented this P2X7 mediated degranulation of the meningeal mast cells as the number of degranulated mast cells dropped two-times to 33.4 ± 5.5%; (*n* = 6; Figures 4F,G). Thus, the H_2_S donor prevented the ATP-induced degranulation of meningeal mast cells.

### NaHS Decreased the Level of Extracellular ATP in Rat Meninges

**Figure 5:**
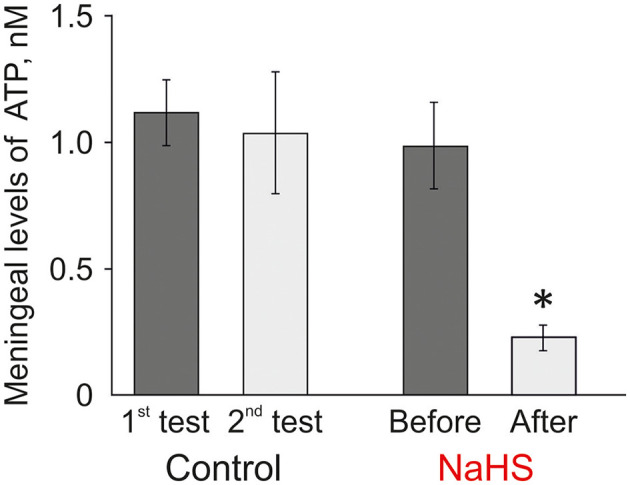
Effect of the H_2_S donor NaHS on the level of extracellular ATP in the meninges in a hemiskull preparation. The ATP level was twice determined in the basic solution in control (interval 20 min) or before and 20 min after incubation in 100 μM NaHS (*n* = 8). *p < 0.05.

Many cells including the meninges (Yegutkin et al., 2016) can spontaneously release ATP to provide its tone in the trigeminovascular system. Next, we analyzed the level of ATP released from rat meninges under control conditions and after incubation of the hemiskull preparation in NaHS. The ATP level in the meninges in control conditions was 1.12 ± 0.13 nM, and after 20 min this level was not significantly changed (1.04 ± 0.24 nM; *n* = 8; Figure 5, left). In the experimental group, the initial ATP value was 0.99 ± 0.17 nM, and after incubation in a solution containing 100 μM NaHS for 20 min, the ATP level significantly decreased to 0.23 ± 0.05 nM (*n* = 8; *p* < 0.05). Based on this data, it can be assumed that H_2_S can affect the transport of ATP through the membrane and reduce the basal level of ATP in meninges.

## Discussion

The main finding of this study is that in meninges, the gaseous transmitter H_2_S exerts the multicomponent protecting action against the powerful nociceptive agent, extracellular ATP. This finding is unusual as H_2_S can itself transiently promote nociceptive firing in trigeminal afferents (Koroleva et al., 2017).

Migraine remains a largely unsolved issue due to still poorly understood pathophysiological mechanisms of headache. In particular, we still not completely characterized a large group of endogenous substances involved in triggering migraine attacks and molecules that can prevent this disabling condition. The novel members of expanding family of gaseous transmitters represent a group of such molecules which may exhibit either pro- or anti-nociceptive effects in migraine. One well-established migraine promoting gas is NO which has multiple vascular and neuronal targets in the trigeminovascular system (Messlinger et al., 2000, 2020; Marone et al., 2018). Another gaseous transmitter, carbon monoxide (CO) also recently emerged as a novel trigger of migraine (Arngrim et al., 2014). H_2_S only recently attracted attention as a gas implicated in migraine (Koroleva et al., 2017; Teicher et al., 2017). According to Teicher et al. (2017), H_2_S can interact with NO to produce the nociceptive effect in trigeminal neurons * via* its product nitroxyl (HNO).

The peripheral part of the trigeminal nerve in the meninges plays a key role in the pathogenesis of migraine pain (Olesen et al., 2009; Schueler et al., 2014) and represents a potential target for the action of endogenous pro- and anti-nociceptive agents. Extracellular ATP and its metabolites, mediating vasodilation of intracranial vessels were previously proposed as key players in the pathogenesis of migraine (Burnstock, 1981). In our previous study, we identified also the neuronal targets of ATP in meninges showing that ATP increased the frequency of action potentials in the trigeminal nerve fibers by direct activation of the P2X3 subunit-containing receptors (Yegutkin et al., 2016). Consistent with this, here we also showed that the P2X3 agonist α,β-meATP excited a large fraction of isolated rat trigeminal neurons in agreement with previous observations on the expression of these pain-related P2X3 receptors in sensory neurons (Wirkner et al., 2007). Moreover, our recent studies indicated that ATP causes mast cell degranulation with subsequent release of active pro-inflammatory agents, particularly serotonin, which can have its strong effect on meningeal nerve endings (Koroleva et al., 2019). Thus, the pain-producing action of ATP in meninges likely includes several direct and indirect effects involving local immune cells.

Recently discovered endogenous gasotransmitter H_2_S can play both pro- and anti-nociceptive effects in different tissues, mediated through various cellular targets (Matsunami et al., 2009; Okubo et al., 2012; Di Cesare Mannelli et al., 2017; Melo et al., 2019; Xu et al., 2019). This gas is generated by several enzymes including CBS. In different types of sensory neurons, including trigeminal ganglia neurons, the high expression of CBS was detected (Xu et al., 2009; Feng et al., 2013). Further studies indicated that CBS expression increased during inflammation paralleled with enhanced neuronal excitability, mainly *via* suppression of potassium currents (Miao et al., 2014). Consistent with the pro-nociceptive action of H_2_S, in our previous study, we observed the transiently increased spiking of the trigeminal nerve in response to the donor of H_2_S NaHS (Koroleva et al., 2017).

The antinociceptive or pronociceptive effects of H_2_S are dependent on its concentration: the low doses of this gasotransmitter contribute to the reduction of pain, whereas H_2_S in high doses can exert even a pro-nociceptive action (Guo et al., 2020). Interestingly, both activating and inhibitory effects of the H_2_S donor Na_2_S has been shown in neurons of the spinal nucleus of the rat trigeminal nerve (Teicher et al., 2017) suggesting that this gaseous transmitter can exert both pro- and anti-nociceptive effects also in the trigeminovascular system implicated in migraine. Thus, after dural applications of Na_2_S, they also found a short-lasting stimulatory (and sometimes inhibitory) effect this H_2_S donor in medulla neurons and suggested TRPA1 channels for the nociceptive action of H_2_S interacting with NO to produce HNO. In our previous study, by direct recordings of spikes from dural nerves, we found that the firing was preferentially mediated by activation of TRPV1 receptors (Koroleva et al., 2017). Notably, TRPV1 and TRPA1, two members of the TRP receptors family are often co-localized (Nielsen et al., 2018). Moreover, there are data that these channels can be heteromerized (Fischer et al., 2014; Weng et al., 2015) suggesting that H_2_S might act in employing these channels complex. Recently, Roa-Coria et al. (2019) proposed that not only TRPV1 and TRPA1 but also TRPC receptors were implicated in the action of H_2_S.

Given that both ATP and H_2_S are endogenous compounds with a clear pro-nociceptive for ATP but more controversial (either pro- or anti-nociceptive) action of H_2_S, it is of interest to explore their potential crosstalk. The functional interactions in the modulation of pain signaling between ATP and NO, another gaseous transmitter implicated in migraine have already previously been shown. Thus, the selective P2X3/P2X2/3 antagonist A-317491 reduced the formation of NO by inhibiting the specific gas generating enzyme nNOS in the neuropathic mouse pain model (Ohnishi et al., 2009). However, the possible interactions between purinergic and H_2_S signaling pathways were not studied yet.

In this study, we showed that the application of NaHS on meningeal afferents prevented the increase of action potentials in response to ATP application. One potential mechanism for such action might the indirect involvement of TRPV1 channels which are the target for the stimulatory action of H_2_S (Koroleva et al., 2017). It has been shown that 33–58% of all trigeminal ganglia neurons co-express P2X3 and TRPV1 and both potentiating and inhibiting interactions between P2X3 and TRPV1 were shown in nociceptors (Saloman et al., 2013). Thus, in isolated neurons, the pre-activation of TRPV1 receptors decreased the subsequent activation of P2X3 receptors due to the inhibitory interactions of the C-termini of P2X3 and TRPV1 proteins (Stanchev et al., 2009; Saloman et al., 2013). Here, in a more complex structure such as the hemiskull preparation, we show that the prior stimulation of TRPV1 receptors by the specific agonist capsaicin did not prevent the nociceptive effects of ATP on meningeal afferents. These data suggest that the depressant action of H_2_S on ATP nociception was not related to TRPV1-mediated inactivation of P2X3 receptors and most likely, mediated by independent signaling cascade initiated by this gaseous transmitter. To reveal the receptor mechanism of NaHS action, isolated trigeminal ganglion neurons were analyzed using electrophysiological patch-clamp methods and calcium imaging. We showed that the application of NaHS decreased fast and mixed currents activated by α,β-meATP which is consistent with data obtained in the meninges indicating the action of H_2_S on P2X3 subunit-containing receptors. These data obtained in voltage-clamped neurons suggest that the H_2_S donor directly affects the function of ionotropic P2X3 receptors as a plausible explanation for the prevention of the pro-nociceptive effect of ATP in peripheral trigeminal afferents.

Recently, the role of Kv7 channels in modulating neuronal excitability was proposed in pain processing (Zheng et al., 2013; Busserolles et al., 2016). Inorganic and slow-releasing H_2_S donors (including the natural allyl-isothiocyanate and its derivatives) were shown to activate Kv7 channels and were effective in animal models of neuropathic pain induced by paclitaxel or oxaliplatin (Di Cesare Mannelli et al., 2017; Lucarini et al., 2018). Thus, the Kv7 channel, in our model, maybe an additional target of anti-nociceptive effects of H_2_S along with inhibition of ATP mediated signaling.

Sulfur in the H_2_S molecule exists in the lowest oxidation state (-2). Therefore, H_2_S is rather a reductant and can be oxidized in the corresponding environment. As a result, H_2_S can elicit its biological effects *via* several chemical reactions. The chemical reduction of protein disulfide bonds by H_2_S was shown (Sitdikova et al., 2010; Pálinkás et al., 2015; Vasas et al., 2015). The reducing action of H_2_S is responsible for its effects on Ca^2+^-activated K^+^ channels and NMDA-receptors (Abe and Kimura, 1996; Sitdikova et al., 2010; Kimura, 2016). Recently, several studies revealed that H_2_S can react with protein thiol groups and form protein persulfides resulting in the functional changes of the target proteins (Mustafa et al., 2009). However, the conversion of R–SH to R–SSH is associated with oxidation, therefore, the oxidation of H_2_S to per-/poly-sulfide or the oxidation of the target cysteine to sulfenic acid or disulfide is necessary for this reaction (Greiner et al., 2013).

Ionotropic P2X receptors activity can be regulated by oxidative stress. It has been shown that H_2_O_2_ may modulate the P2X channel function through the direct oxidation of the cysteine moieties (Coddou et al., 2009). Additionally, the reducing agent dithiothreitol applied intracellularly decreased the sensitivity of the P2X2 receptor to ATP (Nakazawa et al., 2003). Based on these data, we can suggest that the inhibitory effects of H_2_S on P2X3/2 receptors are mediated by its reducing action on disulfide bonds of the channel protein, probably from the intracellular side of the membrane.

Meningeal mast cells play an important role in the pathogenesis of migraine pain due to the pro-inflammatory mediators contained in these immune cells (Levy et al., 2007; Wang et al., 2013). In particular, serotonin released in meninges after degranulation of mast cells by ATP powerfully activates nerve terminals *via* ligand-gated C-loop 5-HT3 receptors (Koroleva et al., 2019). Recent work indicated that H_2_S can prevent mast cell degranulation in a mouse model of asthma (Roviezzo et al., 2015). In models of pruritus and acute skin inflammation, a donor of H_2_S significantly reduced the level of histamine and attenuated C48/80-induced itching probably due to the stabilizing of mast cells (Rodrigues et al., 2017). But H_2_S effects were not tested in meninges where mast cells may have region-specific properties making them different from mast cells in skin or lung tissues.

In our experiments, incubation of meninges with NaHS, resulting in a reduced number of degranulated mast cells exposed to ATP and P2X7 receptor agonist, BzATP. A possible explanation for this effect is that, like inhibitory action of P2X3 receptors, H_2_S inhibits activation of P2X7 receptors thus preventing ATP-induced mast cell degranulation. P2X7 subtype was identified in meningeal mast cells as the main receptor mediating ATP-induced activation of these immune cells (Nurkhametova et al., 2019) and NaHS in our experiments, reduced the number of degranulated cells induced by P2X7 receptor agonist, BzATP. Stabilization of mast cells by H_2_S should prevent serotonin release and activation of the trigeminal nerve, thus preventing this powerful mechanism of peripheral nociceptive signaling in migraine (Kilinc et al., 2017; Koroleva et al., 2019).

Previously, the inhibitory action of H_2_S on P2X7 receptors was hypothesized in a rat model of stroke associated with local inflammation. In that study, H_2_S decreased the inflammatory response down-regulating P2X7 receptors in microglia (Zhao et al., 2017). NaHS could reduce the expression of the P2X7 receptor, decrease membrane permeability, and increase the cell viability in rat microglia injured by ATP. The precise mode of action of H_2_S on the expression and function of the P2X7 receptor should be clarified in further studies.

Finally, among potential targets for H_2_S in the meninges, we found the action of this gaseous transmitter on the level of endogenously produced extracellular ATP. Enhanced release of ATP was observed in the model of migraine with aura (Karatas et al., 2013). ATP release can take place through gap junctions or pannexins, as well as *via* P2X7 receptor channels (Dubyak, 2006). The basic level of ATP in the bulk solution in the meninges was in a nanomolar range of concentrations consistent with previous studies (Yegutkin et al., 2016). Here, we found that NaHS treatment significantly decreased the level of extracellular ATP in meninges. This finding should further contribute to the protective action of H_2_S against the nociceptive signaling by endogenous ATP.

## Conclusion

We analyzed the crosstalk in signaling between two endogenous messengers ATP and H_2_S in the rat trigeminal system implicated in migraine pain. We demonstrated here that H_2_S prevented ATP-induced activation of the trigeminal nerve fibers thus showing the anti-nociceptive effect. One mechanism of this effect is a decrease in membrane currents through P2X3 receptors along with suppression of ATP-induced calcium signals in trigeminal ganglion neurons. Also, H_2_S reduced the ATP levels in the meninges and prevented ATP-induced mast cell degranulation. These data indicate a novel multicomponent pain-preventing role of H_2_S, which is expected to be especially pronounced in conditions of neuroinflammation associated with enhanced release of ATP. The promotion of H_2_S synthesis within the trigeminovascular system, might, therefore, be a novel therapeutic approach for the treatment or prevention of migraine pain.

## Data Availability Statement

The raw data supporting the conclusions of this article will be made available by the authors, without undue reservation.

## Ethics Statement

The animal study was reviewed and approved by the Local Ethics Committee of Kazan Federal University (protocol No. 8 dated 05.05.2015) and the Committee for the Use of Animals of the University of Eastern Finland (licenses EKS-004-2014 and EKS-002-2017).

## Author Contributions

KK contributed to data collection, analysis, interpretation, and writing the manuscript. RGiniatullina, EE, and AM contributed to data collection and analysis. GS and RGiniatullin contributed to the study design and supervision, writing the manuscript, and the final editing. All authors contributed to the article and approved the submitted version.

## Conflict of Interest

The authors declare that the research was conducted in the absence of any commercial or financial relationships that could be construed as a potential conflict of interest.
