# Supplementary material for: Protective Effects of Hydrogen Sulfide Against the ATP-Induced Meningeal Nociception
Source: Front Cell Neurosci. 2020 Sep 2;14:266. doi: 10.3389/fncel.2020.00266 (PMC7492747; doi:10.3389/fncel.2020.00266)
Supplement: Supplementary file 1 [file Data_Sheet_1.PDF]

Supplementary Material

1.1 Supplementary Figures

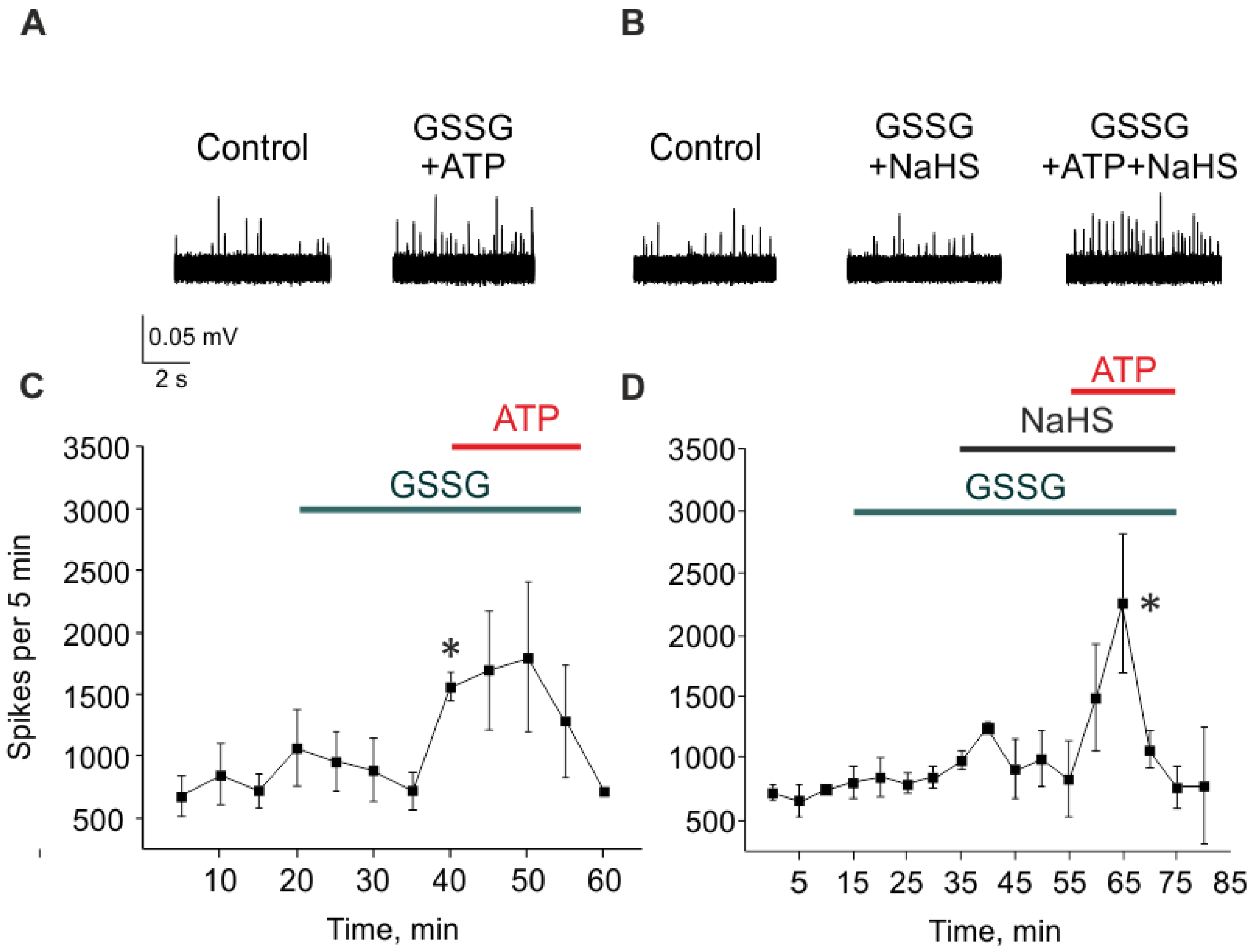

Figure S1. Oxidized glutathione eliminates the inhibitory effects of NaHS on pro-nociceptive action of ATP in trigeminal afferents. A. Example traces of action potentials in the trigeminal nerve in control, after application of oxidized glutathione (GSSG, 1 mM) plus ATP (100 µM); B. Example traces of action potentials in the trigeminal nerve in control, after application of oxidized glutathione (GSSG, 1 mM) plus NaHS (100 µM), and ATP after preincubation in GSSG+NaHS; C. The frequency of action potentials during application of ATP (100 µM) after preincubation with GSSG (1 mM) (n=3); D. The frequency of action potentials during incubation in GSSG (1 mM), GSSG+NaHS and ATP in the presence of GSSG+NaHS (n=3); $*p<0.05$.

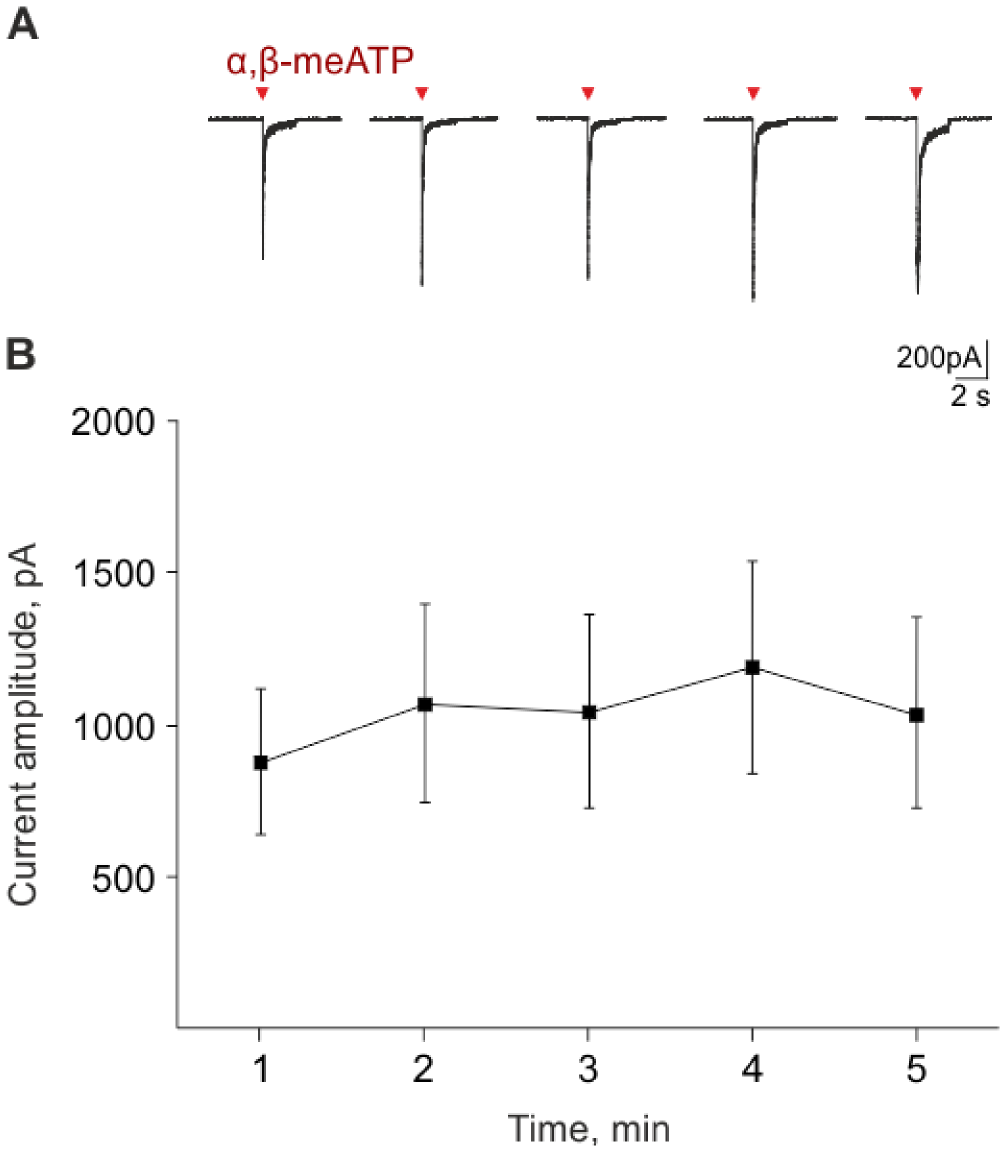

Figure S2. α,β-meATP induced currents in trigeminal ganglion neurons. A. Examples of α,β-meATP -induced currents during repetitive 5 min interval application of agonist. B. Average amplitude of α,β-meATP -induced currents in 5 repetitive application of agonist (7 cells; n=4)

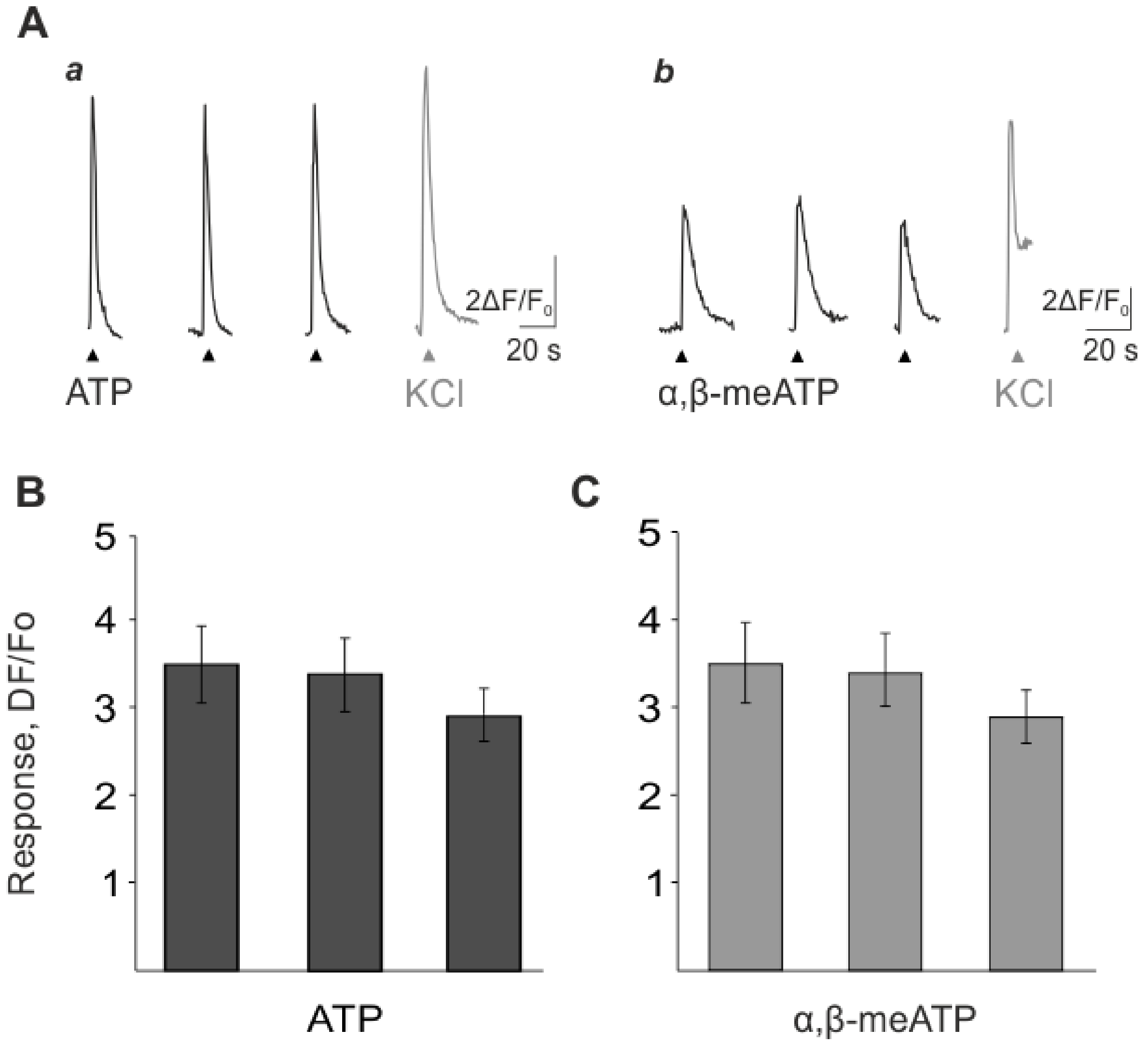

Figure S3. ATP/ α,β-meATP -induced calcium signals in isolated trigeminal neurons. A. Examples of calcium signals induced by 3 consequent 5 min interval applications of 100 µM ATP (a), or 20 µM α,β-meATP (b). KCl was applied to distinguish neurons from glial cells. B. Histograms showing the amplitudes of calcium signals in response to repetitive applications of ATP (100 µM) (61 cells; n=3;). C. Histograms showing the amplitudes of calcium signals in response to repetitive applications of α,β-meATP (20 µM) (33 cells; n=3).
